# Supplementary material for: Testing Strategies of the In Vitro Micronucleus Assay for the Genotoxicity Assessment of Nanomaterials in BEAS-2B Cells
Source: Nanomaterials (Basel). 2021 Jul 27;11(8):1929. doi: 10.3390/nano11081929 (PMC8399994; doi:10.3390/nano11081929)
Supplement: Supplementary file 1 [file nanomaterials-11-01929-s001.zip › nanomaterials-1292137-supplementary.pdf]

Supplementary Materials

Testing Strategies of the In Vitro Micronucleus Assay for the Genotoxicity Assessment of Nanomaterials in BEAS-2B Cells

Tereza Cervena [1,2,†], Andrea Rossnerova [3,†], Tana Zavodna [3], Jitka Sikorova [3], Kristyna Vrbova [1], Alena Milcova [3], Jan Topinka [3] and Pavel Rossner, Jr. [1,*]

[1] Department of Nanotoxicology and Molecular Epidemiology, Institute of Experimental Medicine CAS, Videnska 1083, 142 20 Prague 4, Czech Republic; tereza.cervena@iem.cas.cz (T.C.); kristyna.vrbova@iem.cas.cz (K.V.)

[2] Department of Physiology, Faculty of Science, Charles University, 128 44 Prague 2, Czech Republic

[3] Department of Genetic Toxicology and Epigenetics, Institute of Experimental Medicine CAS, Videnska 1083, 142 20 Prague 4, Czech Republic; andrea.rossnerova@iem.cas.cz (A.R.);
tana.zavodna@iem.cas.cz (T.Z.); jitka.sikorova@iem.cas.cz (J.S.); alena.milcova@iem.cas.cz (A.M.); jan.topinka@iem.cas.cz (J.T.)

* Correspondence: pavel.rossner@iem.cas.cz; Tel.: +420-241-062-763; Fax: +420-241-062-785

† These authors contributed equally to this work.

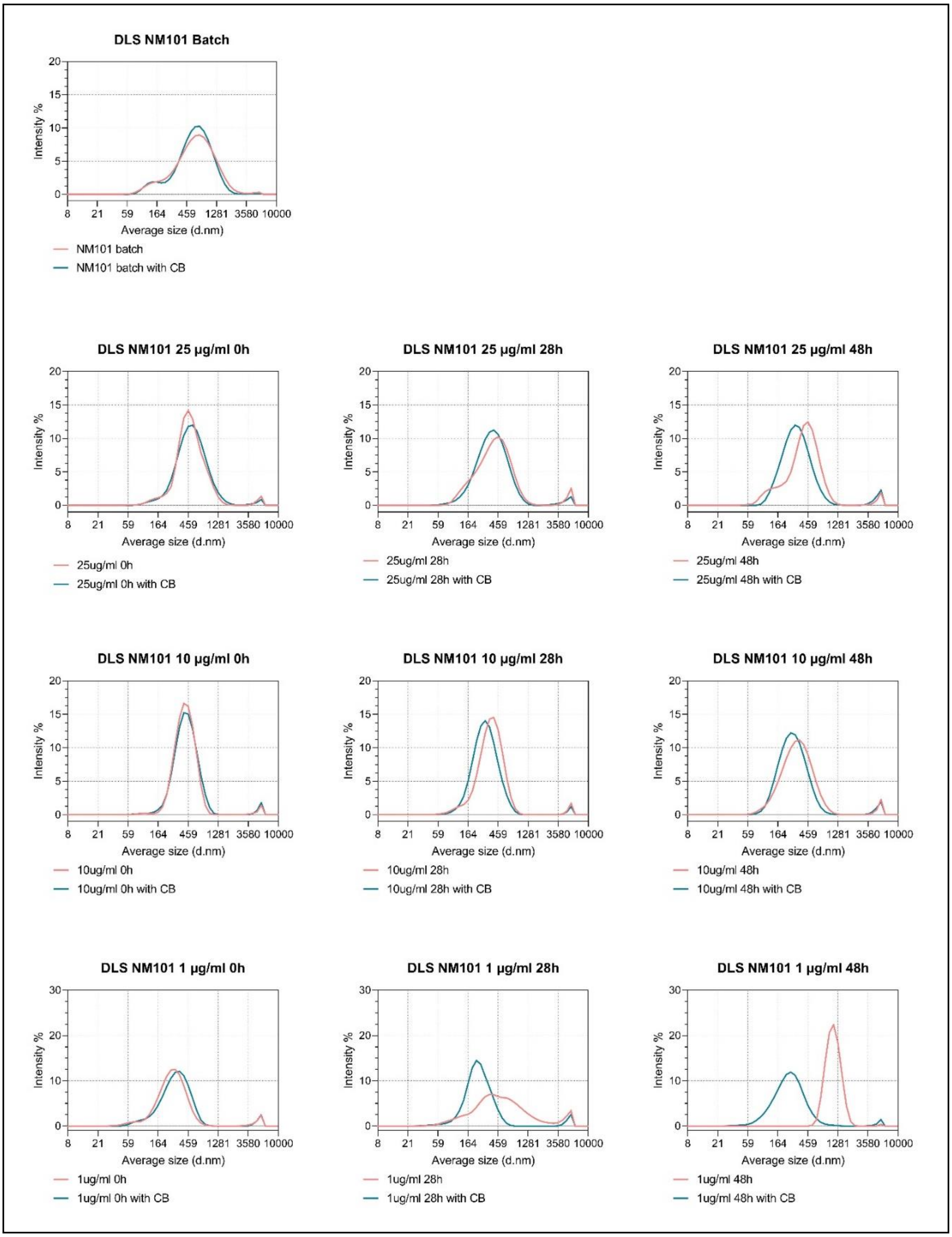

Figure S1: Intensity-based DLS data for TiO_2 NM101

Dynamic light scattering (DLS) measurement of NM after preparation in diH_2O (batch), and in BEGM™ culture medium 0 h, 28 h and 48 h after preparation. NM characterisation in BEGM™ culture medium was done without and with CB (1 µg/ml).

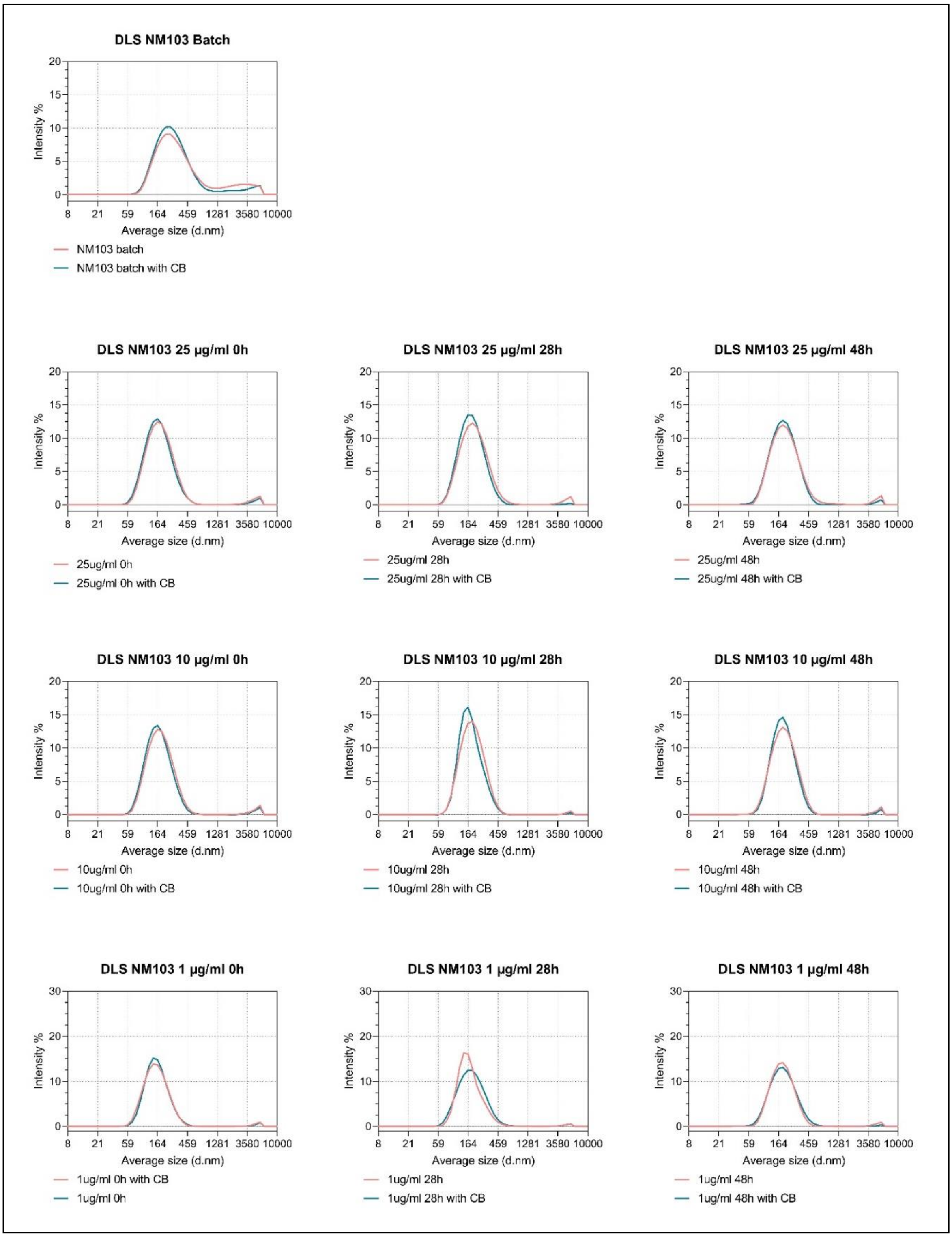

Figure S2: Intensity-based DLS data for TiO_2 NM103

Dynamic light scattering (DLS) measurement of NM after preparation in diH_2O (batch), and in BEGM™ culture medium 0 h, 28 h and 48 h after preparation. NM characterisation in BEGM™ culture medium was done without and with CB (1 µg/ml).

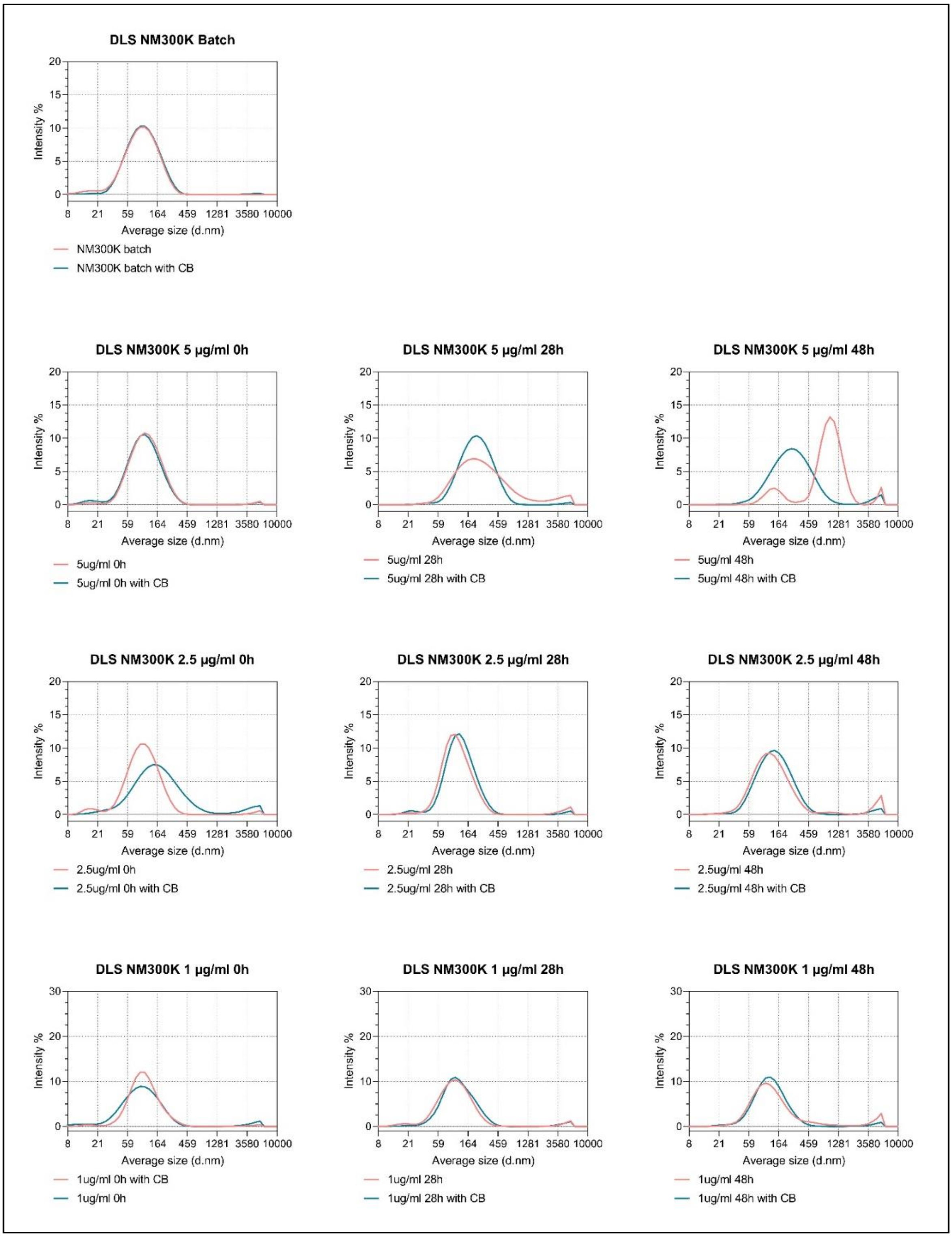

Figure S3: Intensity-based DLS data for Ag NM300K

Dynamic light scattering (DLS) measurement of NM after preparation in diH_2O (batch), and in BEGM™ culture medium 0 h, 28 h and 48 h after preparation. NM characterisation in BEGM™ culture medium was done without and with CB (1 µg/ml).

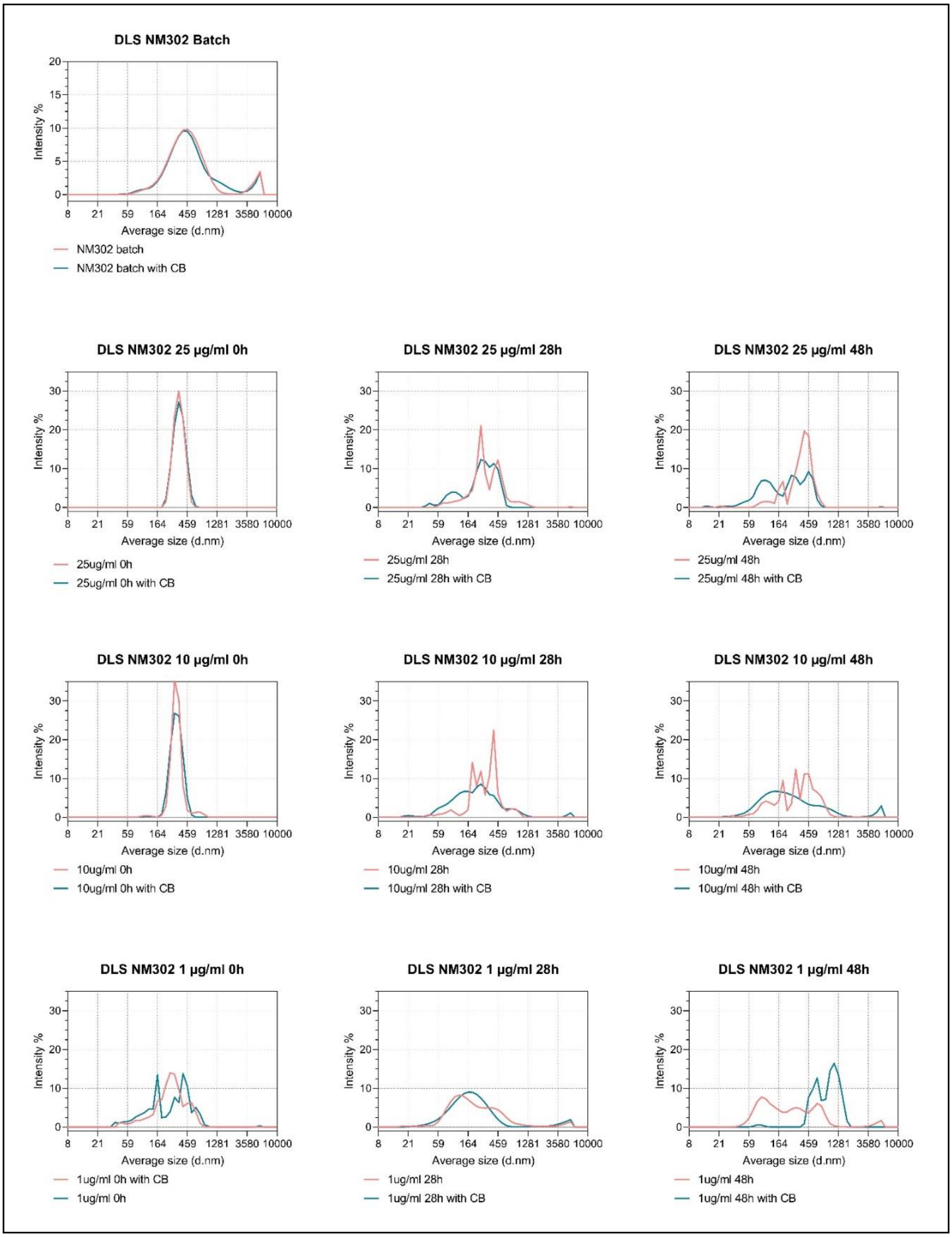

Figure S4: Intensity-based DLS data for Ag NM302

Dynamic light scattering (DLS) measurement of NM after preparation in diH_2O (batch), and in BEGM™ culture medium 0 h, 28 h and 48 h after preparation. NM characterisation in BEGM™ culture medium was done without and with CB (1 µg/ml).

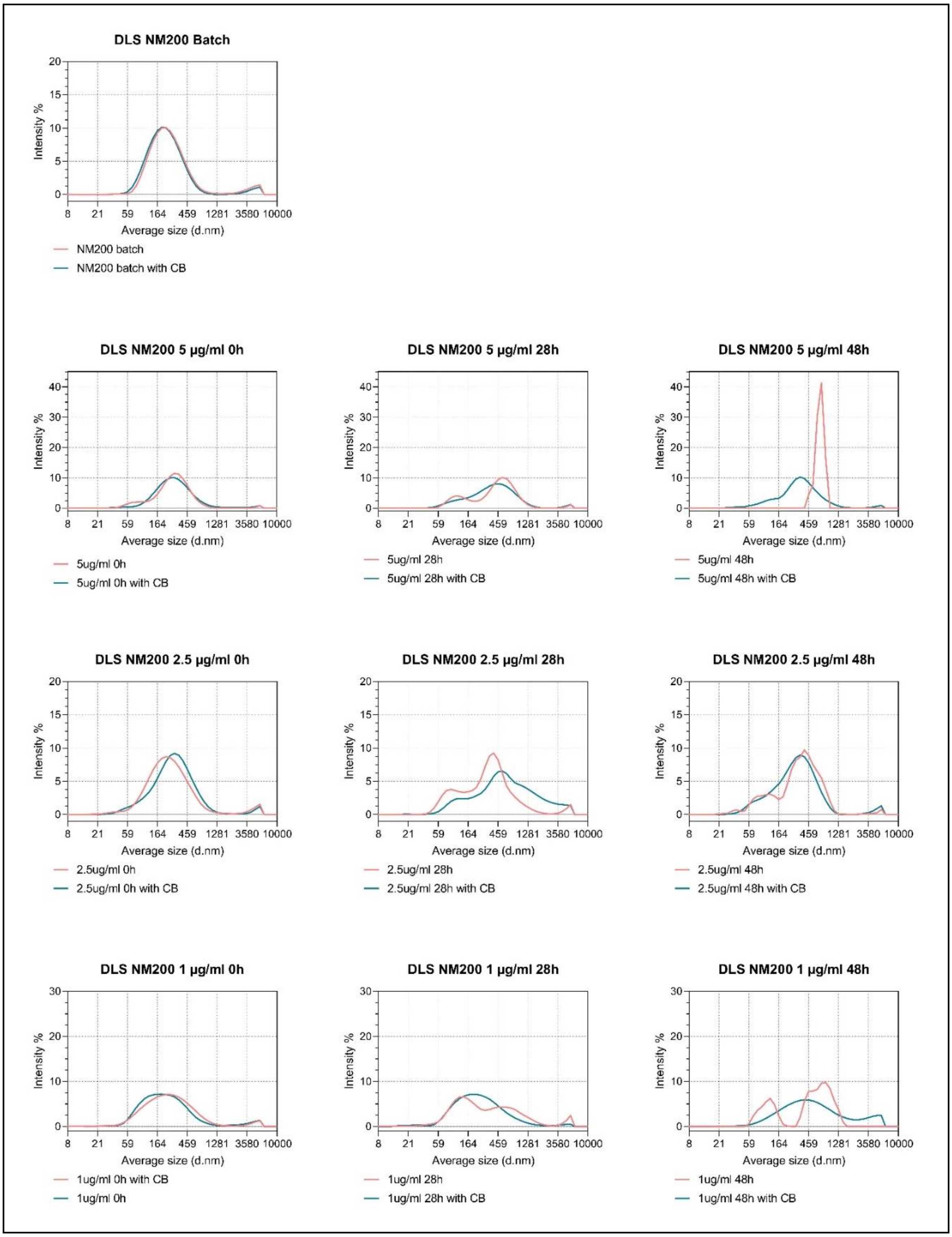

Figure S5: Intensity-based DLS data for SiO_2 NM200

Dynamic light scattering (DLS) measurement of NM after preparation in diH_2O (batch), and in BEGM™ culture medium 0 h, 28 h and 48 h after preparation. NM characterisation in BEGM™ culture medium was done without and with CB (1 µg/ml).
